# Severe Lower Respiratory Tract Infection in Early Infancy and Pneumonia Hospitalizations among Children, Kenya

**DOI:** 10.3201/eid1902.120940

**Published:** 2013-02

**Authors:** Patrick Kiio Munywoki, Eric O. Ohuma, Mwanajuma Ngama, Evasius Bauni, J. Anthony G. Scott, D. James Nokes

**Affiliations:** Author affiliations: KEMRI-Wellcome Trust Research Programme Centre for Geographic Medicine Research–Coast, Kilifi, Kenya (P.K. Munywoki, E.O. Ohuma, M. Ngama, E. Bauni, J.A.G. Scott, D.J. Nokes);; University of Oxford, Oxford, UK (E.O. Ohuma, J.A.G. Scott);; University of Warwick, Coventry, UK (D.J. Nokes)

**Keywords:** respiratory syncytial virus, lower respiratory tract infection, pneumonia, infancy, childhood, wheeze, postdischarge, respiratory infections, hospitalization, viruses, Kenya

## Abstract

Severe lower respiratory tract infection (LRTI) in infants caused by respiratory syncytial virus (RSV) has been associated with later pneumonia hospitalization among children. To determine risk for pneumonia after RSV hospitalization in infancy, we conducted a retrospective cohort analysis of 2,813 infants admitted to a hospital in Kenya and identified readmissions for pneumonia among this group during early childhood (<60 months of age). Incidence of readmission for pneumonia was higher for children whose first admission as infants was for LRTI and who were <3 months of age than for children who were first admitted as infants for non-LRTI, irrespective of RSV status. Incidence of readmission for pneumonia with wheeze was higher for children whose first admission involved RSV compared with those who had non-RSV LRTI. Excess pneumonia risk persisted for 2 years after the initial hospitalization. Close postdischarge follow-up of infants with LRTI, with or without RSV, could help prevent severe pneumonia later in childhood.

## Abstract 2

Close postdischarge follow-up could help prevent future severe respiratory disease.

Pneumonia is a major cause of illness and death among children <5 years of age in sub-Saharan Africa (*1*,*2*), and respiratory syncytial virus (RSV) is the most common viral cause of pneumonia and bronchiolitis in this age group (*3*,*4*). RSV infection in infancy is associated with other long-term respiratory problems (*5*–*10*) and, in one study, with pneumonia (*11*). The magnitude and duration of the increased risk for pneumonia after RSV infection are poorly defined (*12*). In addition, it is not clear whether this association is specific to RSV or whether other causes of lower respiratory tract infection (LRTI) in infancy are also associated with later pneumonia (*11*). A study in The Gambia reported an increased incidence of hospital admission for pneumonia, measurable up to 3 years after discharge (*11*).

We report results of a retrospective cohort analysis of children admitted to a rural district hospital in Kenya using data from a prospective longitudinal clinical surveillance project nested within a health and demographic surveillance system (*13*). The cohort was defined as all infants admitted to the hospital during 9 RSV seasons during 2002–2010; the infants were classified into exposure groups on the basis of the clinical features of LRTI and laboratory diagnosis of RSV at the first admission. The main outcome was readmission to a hospital for pneumonia before the age of 5 years.

## Methods

## Study Population

The study took place in the pediatric wards of Kilifi District Hospital (KDH), a rural hospital on the Indian Ocean coast of Kenya. Since 2001, data on all pediatric admissions and discharges of children at KDH have been prospectively collected in real time through an integrated online data management system in FileMaker Pro (FileMaker, Inc., Santa Clara, CA, USA). Starting from April 16, 2002, we linked admission records to the individual residence status of each child at the date of illness onset within the Kilifi Health and Demographic Surveillance System (KHDSS). The KHDSS covers a population of ≈250,000 living close to the hospital; ≈60% of pediatric admissions of children at KDH originate from KHDSS residents (*13*,*14*). The KHDSS fieldworkers enumerate births, deaths, and migrations (i.e., into, out of, and within the study area) every 4 months. 

Since January 2002, continuous surveillance for RSV has been conducted among all children <60 months of age admitted to KDH for LRTI (*15*). A nasal specimen (nasal wash by bulb or nasopharyngeal aspirate) is collected from each patient by trained medical assistants soon after admission (*15*,*16*) and assayed for RSV antigen by immunofluorescence antibody testing (Imagen Respiratory Syncytial Virus Kit; Oxoid Ltd., Hampshire, UK; or Light Diagnostics Respiratory Viral Screen DFA Kit; Millipore, Temecula, CA, USA), according to the manufacturer’s instructions.

For our study, the cohort was defined as residents of KHDSS admitted to KDH during the first year of life during RSV epidemics from April 16, 2002, through May 31, 2010. We classified the cohort into those who did or did not have LRTI on their first (index) admission. Among those with LRTI, we further subdivided the children on the basis of RSV immunofluorescence antibody test results. Children with LRTI not tested for RSV were examined in preliminary analyses and consolidated into the negative RSV test group. The 3 exposure groups in the cohort were the RSV LRTI group, the other LRTI group, and the non-LRTI group. Infants were excluded if they were admitted on the day of birth; had features of severe malnutrition (i.e., severe wasting, bipedal edema, or mid-upper arm circumference <11 cm); were born with low birthweight (<2.5 kg); had underlying congenital diseases; or remained in the hospital for >2 weeks. Evidence suggests that infants in these categories are at a higher risk for readmission and postdischarge death (*17*).

We used the linked databases of the KDH and KHDSS to determine which cohort members were subsequently readmitted to the hospital or died before their fifth birthday. The primary outcome was readmission to KDH for pneumonia. As secondary outcomes, we examined pneumonia with concurrent wheezing (a marker of hyperreactive airways), all-cause, and nonpneumonia readmissions, as well as all-cause mortality. The Kenya Medical Research Institute Scientific Steering Committee and the National Ethical Review Committee granted ethical approval for this study.

### Definition of Terms

RSV epidemics were defined empirically as periods delimited by weeks in which >1 RSV cases were identified in our hospital surveillance and within which >3 RSV cases were found in any contiguous 3-week period, as described (*15*). Pneumonia was defined as history of cough or difficulty breathing and *>*1 of the following: fast breathing for age (>60 breaths/min if <2 months of age or >50 breaths/min if 2–11 months of age); lower chest wall indrawing; low oxygen saturation (<90%) by pulse oximetry; or inability to feed, prostration, or unconsciousness (*18*). LRTI was ascribed at first admission if the child met the criteria for pneumonia or if the clinician’s discharge diagnosis included pneumonia, asthma, or bronchiolitis.

### Statistical Analysis

Data were analyzed by using STATA version 11.2 (StataCorp, College Station, TX, USA). We used the Student *t* test, Mann-Whitney U test, χ^2^ test, or Fisher exact test as appropriate. Admission to the cohort began at the date of first discharge and continued until the child reached 5 years of age, died, or migrated out of KHDSS, or until December 31, 2010, whichever was earliest. Cohort members who migrated back into the KHDSS were readmitted to the cohort at the date of in-migration. 

Incidence rates were calculated as the number of outcome events among cohort members divided by the sum of child-years at risk within the cohort. Incidence rate ratios (IRRs) for pneumonia readmission between the study groups were estimated by using Poisson regression with Lexis expansion, adjusting for covariates related to the index admission (age, sex, admission to high dependency unit [HDU], geographic sublocation of residence, hospital access, hypoxia [oxygen saturation <90% by pulse oximetry], duration of hospital stay) and the follow-up period (RSV epidemics, age, presence of >1 readmissions for diagnoses other than LRTI). A cutoff of 43 admissions per 1,000 child-years, which was the median incidence of all pediatric admissions to the KDH in 2007, was used to create a binary variable for hospital access by administrative sublocation within the KHDSS.

A multivariable Poisson regression model was developed by using a forward stepwise procedure, rejecting variables with a p value >0.05 in likelihood ratio tests. Risk factors were introduced in descending order of strength of association determined from the univariate analysis. We used a robust variance estimator (Huber-White sandwich estimator) to account for within-person correlation of outcomes. We also performed time-to-event analysis with multiple-failure per person and single failure per person (censoring at first readmission with pneumonia), treating death as a competing risk.

## Results

### Baseline Description at Recruitment

The study recruitment period spanned 9 RSV epidemics lasting a total of 217 weeks. During these weeks, 2,813 infants who met the eligibility criteria were admitted to the hospital; 560 had RSV LRTI, 1,140 had other LRTI, and 1,113 did not have LRTI (non-LRTI group). Of the children included in the other LRTI group, 341 (29.9%) were not tested for RSV. The baseline characteristics for children in the 3 groups are shown in Table 1.

**Table 1:** Baseline characteristics of children in study of pneumonia hospitalizations after severe LRTI in infancy, by study group, at time of first admission in Kilifi District Hospital, coastal Kenya, April 16, 2002–May 31, 2010*

Characteristics	Initial hospitalization		
	RSV LRTI, n = 560	Other LRTI, n = 1,140	Non-LRTI, n = 1,113
Male	296 (52.9)	646 (56.7)	607 (54.5)
Median age, mo (IQR)	**3.7 (1.9–6.6)**	4.6 (2.1–7.7)	4.7 (0.3–8.6)
Children age <3 mo	241 (43.0)	**406 (35.6)**	489 (43.9)
Median hospital stay, d (IQR)	**4 (3–5)**	**3 (2–5)**	4 (2–6)
Malaria†	9 (1.6)	**114 (10.0)**	147 (13.2)
Gastroenteritis	34 (6.1)	**140 (12.3)**	338 (30.4)
Pneumonia with wheeze	95 (17.0)	125 (11.0)	NA
Bacteremia, no./n (%)	**7/545 (1.3)**	33/1,109 (3.0)	33/1,053 (3.1)
Admission to high-dependency unit	**18 (3.2)**	89 (7.8)	79 (7.1)
Good hospital access	326 (59.5)	621 (55.4)	636 (58.6)
Hypoxia	**44 (7.9)**	**95 (8.3)**	17 (1.5)

*Values are no. (%) except as indicated. **Boldface** indicates statistical significance in the respective group relative to the non-LRTI group. RSV, respiratory syncytial virus; LRTI, lower respiratory tract infection; IQR, interquartile range; NA, not applicable. †Indicates blood slide testing positive for malaria parasites.

### Cohort Follow-up and Readmissions

The median durations of follow up (interquartile range) were 40.6 (21.4–57.8), 44.2 (22.0–57.6), and 43.9 (20.6–57.1) months for the RSV LRTI, other LRTI, and non-LRTI groups, respectively. Nine children initially in the non-LRTI group and 16 in the other LRTI group were readmitted with RSV LRTI within first year of life, leading to their crossover to the RSV LRTI group, starting from the date of discharge for the readmission.

The RSV LRTI, other LRTI, and non-LRTI groups contributed 1,781.9, 3,693.8, and 3,550.0 child-years of observation (cyo), respectively; the number of associated readmissions was 231, 419, and 337, respectively. Discharge diagnoses are shown in Technical Appendix Table 1. Pneumonia accounted for 131 (57%) readmissions for the RSV LRTI group, 228 (54%) for the other LRTI group, and 119 (36%) for the non-LRTI group. The numbers of children with only 1 readmission for pneumonia were 58, 173, and 82 for the RSV LRTI, other LRTI, and non-LRTI groups, respectively; the numbers with >2 readmissions were 27, 34, and 15, respectively. A total of 62 (2.2%) children were admitted to the hospital during the follow-up period with laboratory-confirmed RSV infections, 12 (2.1%) in the RSV LRTI group, 17 (1.5%) in the other LRTI group, and 33 (3.0%) in the non-LRTI group. Invasive bacterial pathogens were detected in 8 (1.4%), 12 (1.1%), and 15 (1.3%) children in these groups, respectively.

### Comparison of Readmission Rates between Exposure Groups

Adjusted IRRs comparing rehospitalization for pneumonia by exposure groups are shown in Table 2. The rate of readmission for pneumonia in the RSV LRTI group did not differ from that in the other LRTI group (IRR 1.14, 95% CI 0.85–1.53), even after excluding children who were not tested for RSV (IRR 0.99, 95% CI 0.73–1.34). We combined data for the RSV and other LRTI groups to create an all-LRTI group, consisting of all children with prior exposure to any LRTI; the incidence rate for readmission for pneumonia among these children was significantly higher than for those not exposed to LRTI (non-LRTI group) (p<0.001). The observed effect was modified by age at time of exposure among children whose index LRTI admission occurred at age <3 months (IRR 2.83, 95% CI 1.93–4.15) versus >3 months (IRR 1.39, 95% CI 0.99–1.96). An effect modification by age at the index admission for all LRTI infants was also observed for all-cause readmissions but not for nonpneumonia readmissions (Table 2). The incidence rate relative to non-LRTI was 1.57 (95% CI 1.21–2.04) for children whose first admission occurred in the first 3 months of life and 1.04 (95% CI 0.84–1.30) for older children. Results from the time to first pneumonia readmission analysis accounting for death as a competing risk did not change the interpretation of the associations between LRTI exposure and later pneumonia readmissions; hence, only the multiple-failure regression results are reported.

**Table 2:** Results of multivariable Poisson regression analyses for hospital readmission diagnoses and all-cause mortality among children initially hospitalized during infancy at Kilifi district hospital, coastal Kenya, April 16, 2002–May 31, 2010*

Risk factor	IRR (95% CI)				
	Pneumonia†	Pneumonia with wheeze‡	All readmissions	Nonpneumonia	All-cause mortality
All LRTI§					
Non-LRTI	Referent				
Patient age <3 mo	2.83 (1.93–4.15)	NS	1.57 (1.21–2.04)	0.84 (0.69–1.02)	NS
Patient age >3 mo	1.39 (0.99–1.96)		1.04 (0.84–1.30)		
LRTI					
Non-LRTI	Referent				
RSV LRTI	NS	5.37 (2.66–10.83)	NS	NS	0.42 (0.20–0.90)
Other LRTI		3.50 (1.77–6.94)			1.09 (0.70–1.69)
Patient age					
<3 mo at first admission	Referent				
>3 mo at first admission	NS	1.31 (0.79–2.15)	NS	1.38 (1.11–1.71)	0.88 (0.57–1.35)
Access to hospital					
Poor	Referent				
Good	0.80 (0.62–1.02)	0.58 (0.35–0.98)	0.75 (0.63–0.89)	0.74 (0.60–0.91)	1.76 (1.16–2.67)
Length of hospital stay, d					
<7 d	Referent				
>7 d	1.31 (0.88–1.95)	NS	1.21 (0.94–1.56)	1.11 (0.85–1.46)	2.45 (1.52–3.93)
Admitted to HDU					
No	Referent				
Yes	0.48 (0.27–0.85)	NS	0.64 (0.44–0.94)	0.82 (0.52–1.29)	NS
Age at follow-up, mo					
0–11	Referent				
12–23	0.56 (0.45–0.68)	0.54 (0.35–0.82)	0.67 (0.58–0.77)	0.80 (0.65–0.99)	0.29 (0.17–0.49)
24–36	0.20 (0.14–0.28)	0.28 (0.13–0.60)	0.33 (0.27–0.41)	0.50 (0.39–0.65)	0.11 (0.05–0.25)
48–59	0.05 (0.03–0.09)	0.01 (0.002–0.10)	0.13 (0.10–0.17)	0.23 (0.17–0.30)	0.12 (0.06–0.23)
Admission for non-LRTI					
No	Referent				
Yes	1.92 (1.49–2.47)	1.44 (0.84–2.50)	NS	NS	2.03 (1.32–2.67)
Readmission					
During Jan–Jun	Referent				
During Jul–Dec	NS	NS	NS	NS	0.60 (0.39–0.93)

*Risk factors refer to state at the time of first admission, except the last 3 variables (age group in months, occurrence of >1 non-LRTI admissions, and readmission time), which refer to calendar time during follow-up. IRR, incidence rate ratio; LRTI, lower respiratory tract infection; NS, not statistically significant and thus excluded from the final model for the specified outcome; RSV, respiratory syncytial virus; HDU, high dependency unit. †Defined as history of cough or difficulty breathing and >1 of the following: fast breathing for age (>60 breaths/min if <2 months of age or >50 breaths/min if 2–11 months of age); lower chest wall indrawing; low oxygen saturation (<90%) by pulse oximetry; or inability to feed, prostration, or unconsciousness. ‡Pneumonia with concurrent wheeze. §Combined group of children with RSV and other LRTI. IRRs (95% CIs) for readmission with pneumonia, pneumonia with wheeze, any readmission, nonpneumonia, and all-cause mortality comparing RSV LRTI vs. other LRTI group are 1.14 (0.85–1.53), 1.53 (0.92–2.54), 1.10 (0.89–1.37), 1.07 (0.82–1.39), and 0.39 (0.18–0.82), respectively.

Using non-LRTI children as the baseline group, IRRs for pneumonia with wheeze were 5.37 (95% CI 2.66–10.83) in the RSV LRTI group and 3.50 (95% CI 1.77–6.94) in the other LRTI group. These associations were not modified by age at first admission. IRR for pneumonia with wheeze was 1.53 (95% CI 0.92–2.54) in the RSV LRTI group compared with the other LRTI group; excluding children whose RSV status was not known from the other LRTI group lowered IRR to 1.32 (95% CI 0.78–2.26). The mortality rate was significantly lower for RSV-exposed children compared with the non-LRTI (IRR 0.42, 95% CI 0.20–0.90) and other LRTI (IRR 0.39, 95% CI 0.18–0.82; p = 0.013) groups. The mortality rate increased in areas with good access to the hospital (IRR 1.76, 95% CI 1.16–2.67), and incidence rate of subsequent pneumonia after admission to HDU decreased compared with other categories (IRR 0.48, 95% CI 0.27–0.85) (Table 2).

**Figure 1:**
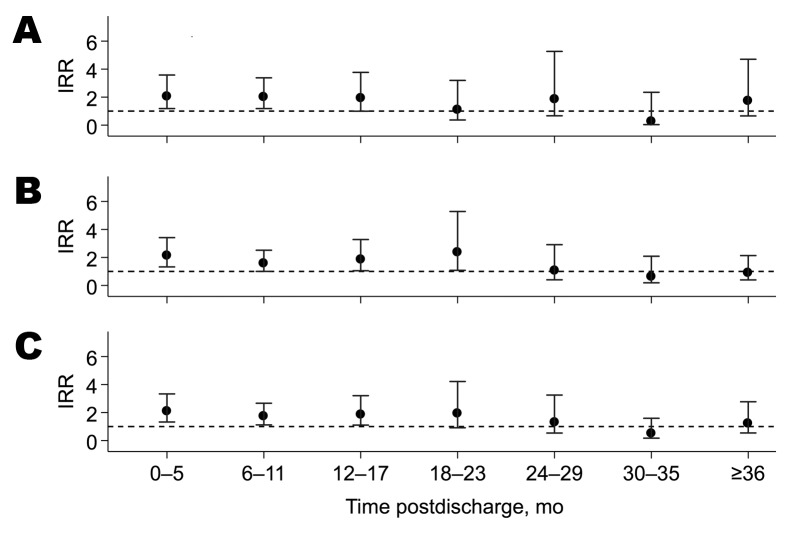
Incidence rate ratios (IRR) for readmission with pneumonia over follow-up time for each of 3 comparisons among children initially admitted during infancy to Kilifi District Hospital, coastal Kenya, April 16, 2002–May 31, 2010. A) Respiratory syncytial virus (RSV) lower respiratory tract infection (LRTI) versus non-LRTI group; B) other LRTI versus non-LRTI group; C) all LRTI (RSV and other LRTI combined) versus non-LRTI group. Error bars indicate 95% CIs.

**Figure 2:**
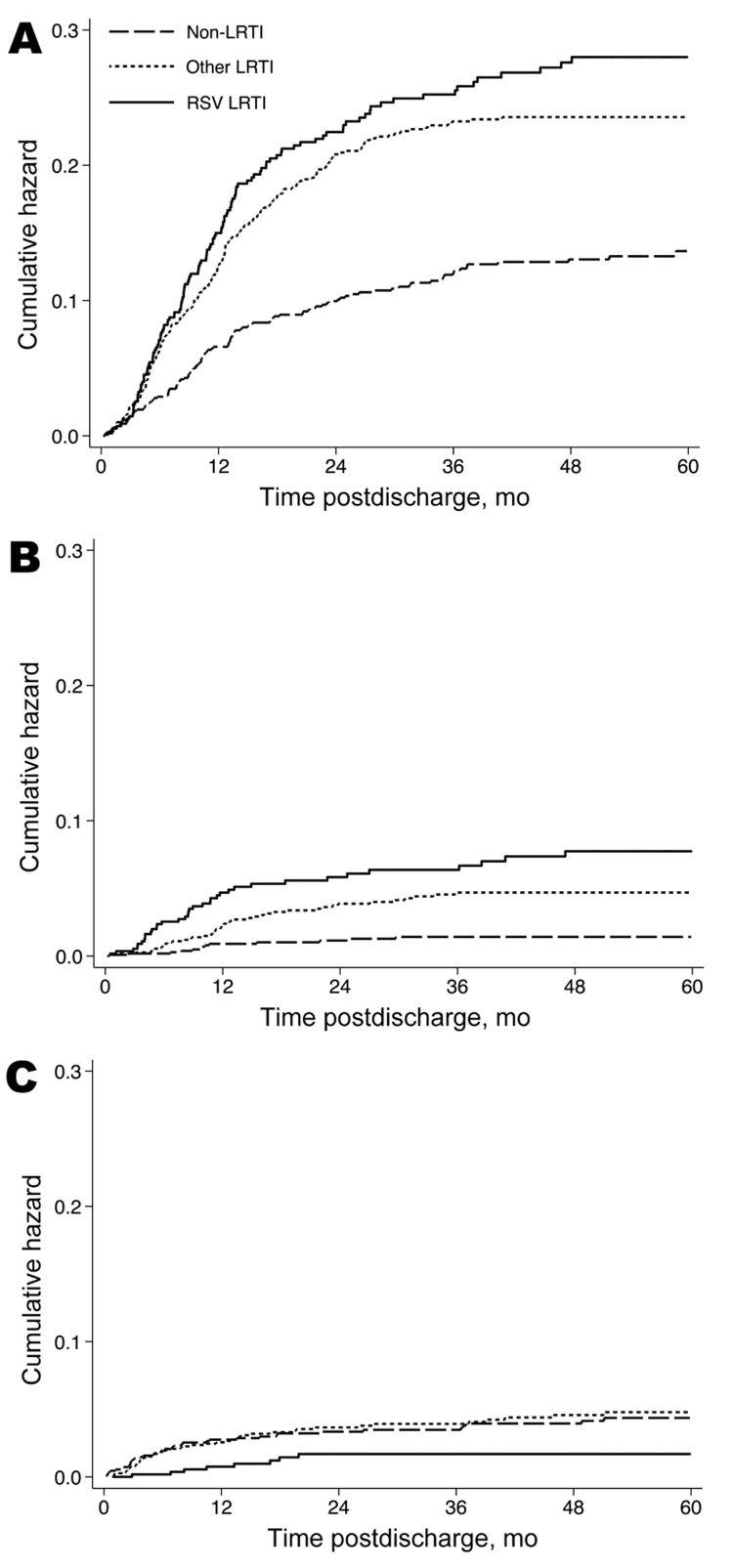
Probability over time of A) readmission for pneumonia, B) readmission for pneumonia with wheeze, and C) death for children with prior respiratory syncytial virus lower respiratory tract infection (LRTI) (solid line), other LRTI (short dashed line), and non-LRTI (long dashed line) during infancy who were hospitalized in Kilifi District Hospital, coastal Kenya, April 16, 2002–May 31, 2010.

Rates of readmission were highest immediately after discharge for all groups, 6/1,000 cyo for the non-LRTI group and 13–15/1,000 cyo for the other LRTI and RSV LRTI groups, and decreased to <2/1,000 cyo at 18–30 months after discharge (Technical Appendix Figure). The differential in the incidence of pneumonia readmissions between LRTI groups and the non-LRTI group over time after discharge is shown as IRRs in Figure 1 and time-to-event profiles in Figure 2. The rate of readmission for pneumonia was higher for the all-LRTI group compared with that for the non-LRTI group up to 2 years after discharge (log-rank test p value <0.001) (Figure 1). The profiles for readmission with pneumonia (Figure 2, panel A) and pneumonia with wheeze (Figure 2, panel B) and for postdischarge death (Figure 2, panel C) show the associations reported in Table 2 to be sustained primarily during the first 12–24 months after discharge.

## Discussion

Using detailed hospital-based data linked to a closely monitored population, we found no difference in the incidence of pneumonia hospitalization among children previously admitted for RSV-associated LRTI in infancy compared with those admitted for non-RSV associated LRTI. However, we did find elevated incidence of readmission for pneumonia among children admitted as infants for LRTI, with or without RSV diagnosis, compared with children admitted as infants for a non-LRTI condition. The magnitude of this association was highest among children admitted in the first 3 months of life and decreased to nonsignificant levels in children >3 months of age at the first admission. Although the association between LRTI during infancy and readmission for pneumonia was unaffected by RSV status at first admission, the association between readmission for pneumonia with wheeze was greater among children whose previous admission was for RSV LRTI (5-fold) compared with children whose previous admission was for LRTI without RSV (3-fold). Only children who were admitted for LRTI at <3 months of age had higher incidence of all-cause readmission compared with those admitted for non-LRTI. The association was lost, with no effect modification by age, when pneumonia admissions at the index admission were excluded; this finding indicates that the later pneumonia was the main driver of the increased incidence of subsequent readmissions and the observed effect modification by age at index admission.

Although this study involves a retrospective analysis, it is based on a large, unique dataset from sub-Saharan Africa in which detailed and consistent data were collected prospectively. KDH is the main inpatient facility in this rural district of Kenya. Previous reports indicate a distance decay in access to KDH, but this would only influence the relative incidence rates between study groups if there was a differential in access by study groups (*14*,*15*). Although Cox regression would have provided a more flexible analysis method, the proportional hazards assumption was violated, and we therefore used Poisson regression with Lexis expansion. Alternative analyses accounting for death as a competing risk yielded no qualitative differences in the study results. To account for the time-dependent incidence (seasonal changes) of exposure to LRTI pathogens, we set the analysis time based on the calendar time in the survival analysis. Seasonal variation in exposure was also minimized by restricting cohort recruitment to RSV epidemic periods only.

Our findings are consistent with the results of similar studies in The Gambia (*11*,*19*) and elsewhere (*6*,*9*,*11*,*20*,*21*). Weber et al. reported a 3-fold increase in incidence of admission for pneumonia or wheezing in children with prior exposure to RSV infection compared with a community of age-matched neighborhood children (*11*). Their study suggested that the duration of increased risk for pneumonia after RSV LRTI in infancy waned by the end of the second year after discharge, a finding duplicated in our study. However, that study did not explore the differential incidence of pneumonia after non-RSV LRTI, nor did it explore the influence of age at first episode of LRTI. Our study also used a hospital-based comparison group for which risk for readmission with pneumonia might have been elevated. 

Prospective studies in Europe and the United States reported that the effects of RSV in infancy on respiratory sequelae decreased sharply during the first year of follow-up and that, by 5 years after RSV infection, the association is insignificant (*11*,*20*,*21*). Other studies have reported persistence of increased respiratory disease up to 7 years (*22*) and 11 years (*8*) after RSV infection but not at 18 years after infection (*5*).

A second observational, age-matched, case–control study in The Gambia examining later lung problems in children after childhood (<5 years of age) admission for severe pneumonia reported inconclusive results (*19*). Even though the odds of lung disease were higher among the childhood pneumonia case-patients compared with controls (odds ratio 2.93, 95% CI 0.69–12.48), the study had severe limitations, including small sample size and potential bias (only 68 of 190 possible cases were traced).

Our findings suggest that, in addition to RSV infection, other etiologies of LRTI are associated with subsequent hospital admissions for pneumonia or wheeze. We offer 2 possible explanations for this. First, children with an inherent predisposition to subsequent pneumonia or wheezing episodes, such as those with smaller airways (*23*–*26*) or with atopy (*27*), may also be more susceptible to viral LRTI in early infancy. Second, viral LRTI in early infancy could lead to structural lung damage or immune paresis that causes further pneumonia episodes with or without wheeze. The latter explanation has been reported for reactive airway disease (mainly manifested as wheeze) in relation to RSV predisposition (*28*–*32*).

In our study, RSV infection appeared to protect against death, but this conclusion is best explained in reverse. In the comparison group, children admitted to a hospital for conditions other than RSV were more likely to die subsequently than were children admitted for RSV. However, unlike some other viruses that cause severe conditions, RSV causes a highly infectious disease that affects most children and does not associate specifically with markers of chronic ill health. A similar phenomenon has been observed with malaria parasitemia, which is associated with a reduced postdischarge mortality rate when compared with admissions for other conditions (*17*).

Findings of an increased mortality rate among children residing in areas with good access to KDH, as well as decreased incidence of subsequent pneumonia following admission to HDU, were unexpected and counterintuitive. Good access was mainly in urban and periurban administrative areas of Kilifi; because urbanization is associated with high HIV prevalence, some of the access-to-care effect may be attributable to HIV/AIDS, but our data lacked HIV results for all admissions to check this effect. The protective association between HDU treatment and later pneumonia may be explained because the HDU has no ventilators and mainly admits children with a nonrespiratory severe illness and because nonrespiratory disease does not have an association with later pneumonia.

We report that hospitalizations for severe LRTI in early infancy in Kenya are associated with increased risk for subsequent pneumonia. The phenomenon has been observed for RSV-associated severe LRTI but not for non-RSV severe LRTI. This raises questions about the underlying cause and, on a practical level, alerts clinicians that a child with LRTI in the first 3 months of life is at risk for readmission with severe respiratory disease over the period of 1–2 years after discharge. Parents of children with LRTI-related hospital admissions during infancy should be advised to be vigilant in the care of the child and to seek medical advice rapidly in the event of further respiratory symptoms. An effective outpatient follow-up for these children throughout early childhood might also be warranted. Larger cohorts and probe studies using interventions against LRTI in infancy (e.g., vaccines) will be pivotal for confirming the causality and the magnitude of the associations observed here and for determining the specificity of the infectious etiologies associated (or not associated) with later episodes of pneumonia.

Technical Appendix Number and crude incidence rates per 1,000 child-years of various diagnoses by study group for readmission to Kilifi District Hospital, coastal Kenya; univariate Poisson regression analysis of risk factors for readmission with pneumonia, pneumonia with wheeze and all-cause mortality; and incidence rates of readmission with pneumonia over follow-up time by study group.

## References

[1] Rudan I, Tomaskovic L, Boschi-Pinto C, Campbell H. Global estimate of the incidence of clinical pneumonia among children under five years of age. Bull World Health Organ. 2004;82:895–903. 15654403 PMC2623105

[2] Black RE, Cousens S, Johnson HL, Lawn JE, Rudan I, Bassani DG, Global, regional, and national causes of child mortality in 2008: a systematic analysis. Lancet. 2010;375:1969–87. 10.1016/S0140-6736(10)60549-1 20466419

[3] Forgie IM, O’Neill KP, Lloyd-Evans N, Leinonen M, Campbell H, Whittle HC, Etiology of acute lower respiratory tract infections in Gambian children: II. Acute lower respiratory tract infection in children ages one to nine years presenting at the hospital. Pediatr Infect Dis J. 1991;10:42–7. 10.1097/00006454-199101000-00009 2003054

[4] Nair H, Nokes DJ, Gessner BD, Dherani M, Madhi SA, Singleton RJ, Global burden of acute lower respiratory infections due to respiratory syncytial virus in young children: a systematic review and meta-analysis. Lancet. 2010;375:1545–55. 10.1016/S0140-6736(10)60206-1 20399493 PMC2864404

[5] Korppi M, Piippo-Savolainen E, Korhonen K, Remes S. Respiratory morbidity 20 years after RSV infection in infancy. Pediatr Pulmonol. 2004;38:155–60. 10.1002/ppul.20058 15211700

[6] Pullan CR, Hey EN. Wheezing, asthma, and pulmonary dysfunction 10 years after infection with respiratory syncytial virus in infancy. Br Med J (Clin Res Ed). 1982;284:1665–9. 10.1136/bmj.284.6330.1665 6805648 PMC1498624

[7] Sigurs N, Gustafsson PM, Bjarnason R, Lundberg F, Schmidt S, Sigurbergsson F, Severe respiratory syncytial virus bronchiolitis in infancy and asthma and allergy at age 13. Am J Respir Crit Care Med. 2005;171:137–41. 10.1164/rccm.200406-730OC 15516534

[8] Stein RT, Sherrill D, Morgan WJ, Holberg CJ, Halonen M, Taussig LM, Respiratory syncytial virus in early life and risk of wheeze and allergy by age 13 years. Lancet. 1999;354:541–5. 10.1016/S0140-6736(98)10321-5 10470697

[9] Singleton RJ, Redding GJ, Lewis TC, Martinez P, Bulkow L, Morray B, Sequelae of severe respiratory syncytial virus infection in infancy and early childhood among Alaska Native children. Pediatrics. 2003;112:285–90. 10.1542/peds.112.2.285 12897275

[10] Sigurs N. A cohort of children hospitalised with acute RSV bronchiolitis: impact on later respiratory disease. Paediatr Respir Rev. 2002;3:177–83. 10.1016/S1526-0542(02)00191-4 12376053

[11] Weber MW, Milligan P, Giadom B, Pate MA, Kwara A, Sadiq AD, Respiratory illness after severe respiratory syncytial virus disease in infancy in The Gambia. J Pediatr. 1999;135:683–8. 10.1016/S0022-3476(99)70085-5 10586169

[12] Bont L, Aalderen WM, Kimpen JL. Long-term consequences of respiratory syncytial virus (RSV) bronchiolitis. Paediatr Respir Rev. 2000;1:221–7. 10.1053/prrv.2000.0052 12531083

[13] Scott JA, Bauni E, Moisi JC, Ojal J, Gatakaa H, Nyundo C, Profile: the Kilifi Health and Demographic Surveillance System (KHDSS). Int J Epidemiol. 2012;41:650–7. 10.1093/ije/dys062 22544844 PMC3396317

[14] Moïsi JC, Gatakaa H, Noor AM, Williams TN, Bauni E, Tsofa B, Geographic access to care is not a determinant of child mortality in a rural Kenyan setting with high health facility density. BMC Public Health. 2010;10:142. 10.1186/1471-2458-10-142 20236537 PMC2848200

[15] Nokes DJ, Ngama M, Bett A, Abwao J, Munywoki P, English M, Incidence and severity of respiratory syncytial virus pneumonia in rural Kenyan children identified through hospital surveillance. Clin Infect Dis. 2009;49:1341–9. 10.1086/606055 19788358 PMC2762474

[16] Ngama MJ, Ouma B, English ME, Nokes DJ. Comparison of three methods of collecting nasal specimens for respiratory virus analysis. East Afr Med J. 2004;81:313–7. 10.4314/eamj.v81i6.9181 16167679

[17] Moïsi JC, Gatakaa H, Berkley JA, Maitland K, Mturi N, Newton CR, Excess child mortality after discharge from hospital in Kilifi, Kenya: a retrospective cohort analysis. Bull World Health Organ. 2011;89:725–32, 32A. 10.2471/BLT.11.089235 PMC3209982 22084510

[18] Nokes DJ, Okiro EA, Ngama M, White LJ, Ochola R, Scott PD, Respiratory syncytial virus epidemiology in a birth cohort from Kilifi district, Kenya: infection during the first year of life. J Infect Dis. 2004;190:1828–32 and. 10.1086/425040 15499540

[19] Puchalski Ritchie LM, Howie SR, Arenovich T, Cheung YB, Weber M, Moore S, Long-term morbidity from severe pneumonia in early childhood in The Gambia, West Africa: a follow-up study. Int J Tuberc Lung Dis. 2009;13:527–32 . 19335961

[20] Bont L, Steijn M, Van Aalderen WM, Brus F, Th Draaisma JM, Van Diemen-Steenvoorde RA, Seasonality of long term wheezing following respiratory syncytial virus lower respiratory tract infection. Thorax. 2004;59:512–6. 10.1136/thx.2003.013391 15170037 PMC1747053

[21] Schauer U, Hoffjan S, Bittscheidt J, Kochling A, Hemmis S, Bongartz S, RSV bronchiolitis and risk of wheeze and allergic sensitisation in the first year of life. Eur Respir J. 2002;20:1277–83. 10.1183/09031936.02.00019902 12449185

[22] Henderson J, Hilliard TN, Sherriff A, Stalker D, Al Shammari N, Thomas HM. Hospitalization for RSV bronchiolitis before 12 months of age and subsequent asthma, atopy and wheeze: a longitudinal birth cohort study. Pediatr Allergy Immunol. 2005;16:386–92 and. 10.1111/j.1399-3038.2005.00298.x 16101930

[23] Young S, O’Keeffe PT, Arnott J, Landau LI. Lung function, airway responsiveness, and respiratory symptoms before and after bronchiolitis. Arch Dis Child. 1995;72:16–24. 10.1136/adc.72.1.16 7717730 PMC1510966

[24] Young S, Arnott J, O’Keeffe PT, Le Souef PN, Landau LI. The association between early life lung function and wheezing during the first 2 yrs of life. Eur Respir J. 2000;15:151–7. 10.1183/09031936.00.15115100 10678638

[25] Martinez FD, Wright AL, Taussig LM, Holberg CJ, Halonen M, Morgan WJ. Asthma and wheezing in the first six years of life. The Group Health Medical Associates. N Engl J Med. 1995;332:133–8. 10.1056/NEJM199501193320301 7800004

[26] Martinez FD, Morgan WJ, Wright AL, Holberg CJ, Taussig LM. Diminished lung function as a predisposing factor for wheezing respiratory illness in infants. N Engl J Med. 1988;319:1112–7. 10.1056/NEJM198810273191702 3173442

[27] Laing I, Reidel F, Yap PL, Simpson H. Atopy predisposing to acute bronchiolitis during an epidemic of respiratory syncytial virus. Br Med J (Clin Res Ed). 1982;284:1070–2. 10.1136/bmj.284.6322.1070 6802409 PMC1497929

[28] Balfour-Lynn IM. Why do viruses make infants wheeze? Arch Dis Child. 1996;74:251–9 and. 10.1136/adc.74.3.251 8787436 PMC1511409

[29] Openshaw PJ. Potential mechanisms causing delayed effects of respiratory syncytial virus infection. Am J Respir Crit Care Med. 2001;163:S10–3. 11254545 10.1164/ajrccm.163.supplement_1.2011111

[30] Welliver RC, Duffy L. The relationship of RSV-specific immunoglobulin E antibody responses in infancy, recurrent wheezing, and pulmonary function at age 7–8 years. Pediatr Pulmonol. 1993;15:19–27. 10.1002/ppul.1950150104 8419894

[31] Renzi PM, Turgeon JP, Yang JP, Drblik SP, Marcotte JE, Pedneault L, Cellular immunity is activated and a TH-2 response is associated with early wheezing in infants after bronchiolitis. J Pediatr. 1997;130:584–93. 10.1016/S0022-3476(97)70243-9 9108857

[32] Pinto RA, Arredondo SM, Bono MR, Gaggero AA, Diaz PV. T helper 1/T helper 2 cytokine imbalance in respiratory syncytial virus infection is associated with increased endogenous plasma cortisol. Pediatrics. 2006;117:e878–86. 10.1542/peds.2005-2119 16618789

