# Supplementary material for: Severe Lower Respiratory Tract Infection in Early Infancy and Pneumonia Hospitalizations among Children, Kenya
Source: Emerg Infect Dis. 2013 Feb;19(2):223–9. doi: 10.3201/eid1902.120940 (PMC3559052; doi:10.3201/eid1902.120940)
Supplement: Technical Appendix — Number and crude incidence rates per 1,000 child-years of various diagnoses by study group for readmission to Kilifi District Hospital, coastal Kenya; univariate Poisson regression analysis of risk factors for readmission with pneumonia, pneumonia with wheeze and all-cause mortality; and incidence rates of readmission with pneumonia over follow-up time by study group. [file 12-0940-Techapp_s1.pdf]

Article DOI: http://dx.doi.org/10.3201/eid1902.120940

Severe Lower Respiratory Tract Infection in Early Infancy and Pneumonia Hospitalizations among Children, Kenya

Technical Appendix

Technical Appendix Table 1. Number and crude incidence rates per 1,000 child-years of various diagnoses by study group for readmission to Kilifi District Hospital, coastal Kenya

	RSV LRTI cyo = 1,781.93		Other-LRTI cyo = 3,693.78		Non-LRTI cyo = 3,550.0	
Diagnoses	N	Inc	N	Inc	N	Inc
All-cause readmission	231	129.6	419	113.4	337	94.9
Pneumonia	131	73.5	228	61.7	119	33.5
Pneumonia with wheeze	36	20.2	44	11.9	13	3.7
All-cause mortality	8	4.5	45	112.2	40	11.3
Malaria (blood slide positive)	23	12.9	83	22.5	71	20.0
Gastroenteritis	45	25.3	72	19.4	68	19.2
Malnutrition	9	5.1	17	4.6	26	7.3
Bacteraemia	8	4.5	11	3.0	15	4.2
Otitis media	2	1.1	7	1.9	5	1.4
Anaemia	12	6.7	26	7.0	30	8.5
Fits	19	10.7	47	12.7	54	115.2
Others	48	26.9	106	28.7	112	31.5

*cyo, child-years of observation; N, number of admissions; Inc, crude incidence rates per 1,000 cyo.

Technical Appendix Table 2. Univariate Poisson regression analysis of risk factors for readmission with pneumonia, pneumonia with wheeze and all-cause mortality*

Risk factors†	Baseline category	Pneumonia‡ IRR (95% CI)	Pneumonia + wheeze§ IRR (95% CI)	All-cause mortality IRR (95% CI)
RSV LRTI	Non-LRTI group	2.19 (1.57–3.06)	5.52 (2.80–10.85)	0.40 (0.19–0.85)
Other LRTI		1.84 (1.40–2.42)	3.18 (1.68–6.03)	1.08 (0.70–1.66)
Male gender	Female	1.03 (0.81–1.32)	1.33 (0.82–2.15)	1.35 (0.89–2.06)
Age at first admission >3m	≤3 months	0.85 (0.67–1.08)	1.31 (0.80–2.14)	0.74 (0.49–1.11)
Hospital stay, more than 7 days	≤7 days	1.23 (0.83–1.81)	1.25 (0.56–2.78)	2.75 (1.75–4.33)
Locations with good access to hospital	Poorly accessible locations	0.77 (0.60–1.00)	0.53 (0.32–0.89)	1.73 (1.15–2.62)
Admission to HDU	Not admitted in HDU	0.49 (0.28–0.86)	0.33 (0.08–1.35)	1.81 (0.93–3.52)
Low O_2 saturation (hypoxia)	>90% at first admission	1.20 (0.65–2.21)	1.16 (0.40–3.35)	1.57 (0.76–3.26)
Readmitted during RSV epidemic	Outside RSV epidemic	1.25 (1.04–1.50)	1.33 (0.89–1.98)	1.87 (1.22–2.88)
Age group (mo) at follow-up				
12–23 m	0–11 months	0.51 (0.42–0.63)	0.51 (0.33–0.79)	0.25 (0.15–0.41)
24–35 m		0.19 (0.14–0.27)	0.29 (0.14–0.59)	0.09 (0.04–0.22)
36–59 m		0.05 (0.03–0.08)	0.05 (0.01–0.23	0.09 (0.05–0.18)
Had ≥1 non-LRTI readmissions	Never had a non-LRTI admission	1.84 (1.43–2.37)	1.53 (0.92–2.55)	1.74 (1.13–2.70)
Readmission time Jul–Dec	Jan–Jun	0.92 (0.76–1.11)	0.86 (0.58–1.27)	0.51 (0.33–0.79)

*IRR, incidence rate ratio; RSV, respiratory syncytial virus; LRTI, lower respiratory tract infection; HDU, high dependency unit.
†Risk factors refer to state at the time of first admission except the last 4 (RSV epidemic, age group, occurrence of one or more non-LRTI admissions, and readmission time), which refer to events during follow up period.
‡Pneumonia as defined in the main text.
§Pneumonia with concurrent wheezing. IRRs (95% CI) for readmission with pneumonia, pneumonia with wheeze, and all-cause mortality comparing RSV LRTI vs. other LRTI group are 1.19 (0.88–1.161), 1.74 (1.02–2.94), and 0.37 (0.17–0.78), respectively.

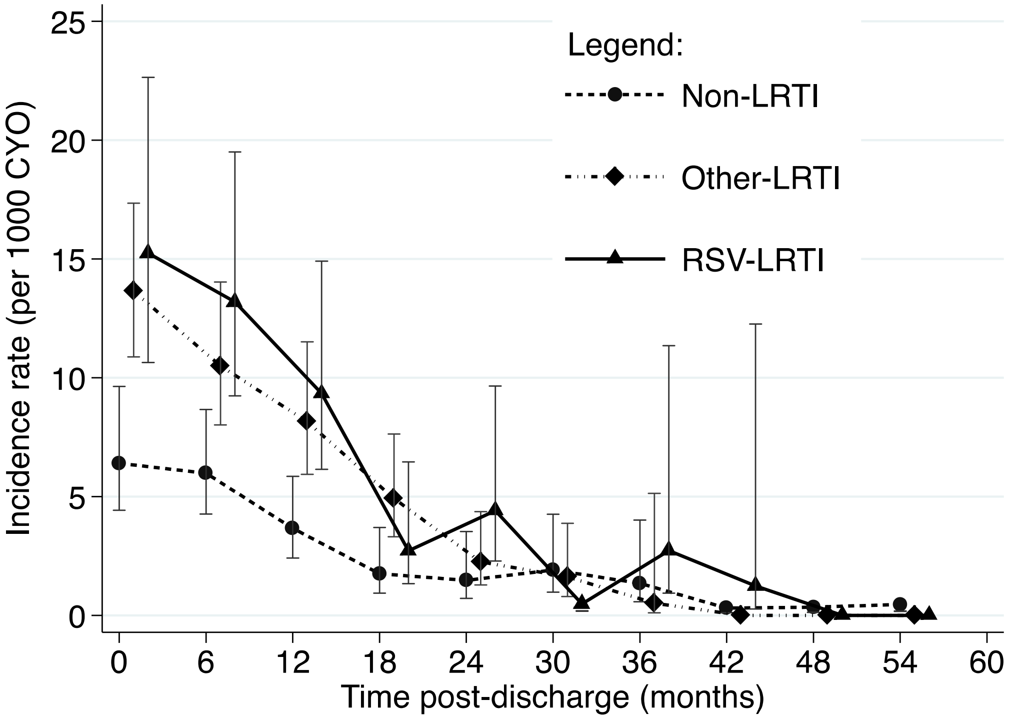

Technical Appendix Figure. Incidence rates of readmission with pneumonia over follow up time by the 3 study groups.
